# The Influence of Devulcanization and Revulcanization on Sulfur Cross-Link Type/Rank: Recycling of Ground Tire Rubber

**DOI:** 10.1021/acsomega.4c06159

**Published:** 2024-09-25

**Authors:** James Robert Innes, Nehnah Siddique, Glen Thompson, Xiaolei Wang, Phil Coates, Ben Whiteside, Hadj Benkreira, Fin Caton-Rose, Canhui Lu, Qi Wang, Adrian Kelly

**Affiliations:** † Polymer IRC, University of Bradford, Richmond Road, Bradford BD71DP, U.K.; ‡ State Key Laboratory of Polymer Materials Engineering, Polymer Research Institute of Sichuan University, Chengdu 610065, China

## Abstract

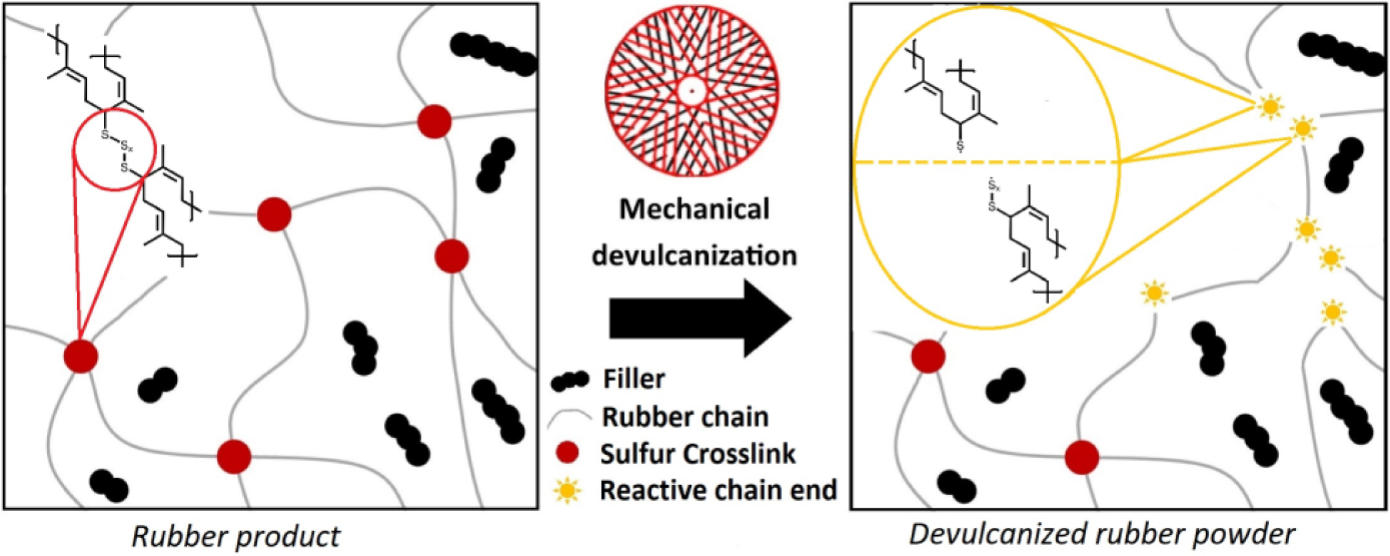

Devulcanized rubber could be a valuable material feedstock to help in the manufacture of sustainable rubber products. However, the differences in the chemistry and structure of devulcanized rubber have limited industrial uptake. This work demonstrates how devulcanization affects the concentration and ratios of mono-, di-, and polysulfidic cross-links. These residual cross-links make devulcanized rubber chemically dissimilar from virgin rubber, affecting (re)vulcanization. The hypothesis that the sulfur rank of revulcanized material can be modified by the sulfur/accelerator ratio was evaluated by two different cure packages. Despite substantial differences in the accelerator/sulfur ratio, both recycled rubber compounds favored the formation of polysulfidic cross-links.

## 1 Introduction

The processes of breaking rubber cross-links (devulcanization) and reforming them (revulcanization) are crucial to controlling the properties of recycled rubber. If we wish to utilize valuable waste feedstocks, such as ground tire rubber (GTR), in the manufacture of new rubber products, then devulcanization and revulcanization must be well understood.

In the case of virgin rubber, the formation of cross-links (vulcanization) is a well-understood process that is controlled by the curatives used, allowing a broad range of properties to be obtained.^1^ This vulcanization process utilizes curatives to produce intermediate radicals capable of initiating the insertion of sulfur cross-links between unsaturated polymer chains. As the vulcanization reaction progresses, these sulfur links reduce in length by a series of reactions. It is well-known that vulcanized products contain a mixture of sulfur cross-link lengths.^2,3^ The ratio of bond types (mono/di/polysulfidic) can be controlled by curatives and vulcanization times and, in turn, affects the resulting material properties. However, how devulcanization processes affect poly-, di-, and monosulfidic cross-links is rarely considered.^4^

Devulcanization techniques (microwave, mechanical, ultrasound, chemical, etc.) are often evaluated in terms of how they affect the cross-link density and gel fraction.^5−16^ Additionally, the Horikx diagram^17^ has been used to demonstrate whether these processes efficiently break sulfur cross-links, rather than breaking the polymer backbone.^18−20^ Devulcanization processes that offer no selectivity between the destruction of the carbon backbone and the sulfur cross-links are referred to as reclaim, whereas sulfur-selective techniques may be referred to as devulcanization.^21^ Yet, little attention is paid to which sulfur bonds are broken by these devulcanization processes.

The reprocessable powders produced by devulcanization are not completely free from cross-links.^5−7,9,11,14,18,22^ Characterization of the residual cross-links is surely important for tailoring the revulcanization cure package, especially if the original product properties are to be reobtained. By measuring the different types of cross-links remaining in recycled rubber, i.e., the ratio of mono/di/polysulfidic linkages, we may seek to understand how to introduce appropriate new cross-links. As such, this article reports on how the solid-state shear milling process for devulcanization of rubber affects the cross-link types of the produced rubber recyclate.

Solid-state shear milling (S3M) is described as a mechanochemical devulcanization technique (however, no chemicals are added) that was developed by researchers at Sichuan University.^23,24^ In this process, rubber materials (and other polymers).^25−27^ can be broken down by two surface-structured steel plates at room temperature, breaking matrix cross-links, resulting in a processable recyclate. Our previous work has shown that this devulcanized powder can be easily reprocessed, either by blending into virgin rubber using a two-roll mill or internal mixer,^28^ or via extrusion with a thermoplastic to produce a thermoplastic vulcanizate.^29^

If the S3M bond-breaking process is selective, then one might anticipate that the bonds with the lowest energy required for cleavage are broken first. These bond energies for S–S, C–S, and C–C bonds are approximately 240, 270, and 345 kJ/mol, respectively,^15,30^ which suggests that bond scission is easier for poly- and disulfidic bonds compared with monosulfidic bonds and the polymer backbone. Yet, Rybinski and Janowska demonstrate wide-ranging activation energies for the thermal decomposition of vulcanizate bonds.^31^ Nevertheless, the resulting properties of the material are dependent on both the cross-link density and the ratio of sulfur types.^32^ For example, polysulfidic bonds provide increased tensile strength (UTS),^33^ which could provide one explanation for why recycled materials tend to have reduced UTS compared with virgin counterparts.^34^ However, the reduction in molecular weight from carbon main chain breakage and the hindrance of crystallization (of natural rubber) must also be considered for the reduced properties of recyclates. If the process is found to be selective for a specific sulfur bond length, then the revulcanization cure package should be tailored to reintroduce those cross-links most affected. Yet, alternative cure packages for revulcanization are not widely reviewed. It has been suggested that half the curatives are required for ground tire rubber compared with virgin rubber,^35^ which was partly supported by our recent work.^28^

It is possible to use a combination of swelling tests and Soxhlet extraction with selective chemical probes to determine the ratios of mono/di/polysulfidic bonds present within cross-linked rubbers.^36,37^ Recently, Choi and Kim modified this technique so that it could be performed with reduced toxicity, demonstrating the ability to infer cross-link types without prior knowledge of the rubber composition.^38^ This may be particularly useful for recycled rubber, where the source material contains a mixture of rubbers of unknown formulation. Valentin et al. used chemical probes to measure the sulfur cross-link ratios of commercial ground tire rubber (GTR).^4^ They found that 38% of the linkages were either C–S or C–C cross-links and also suggested that monosulfidic linkages cannot be broken without chain scission. Additionally, they used ^1^H DQ-NMR to establish that the rubber fraction of a 2.1 mm crumb rubber contained 50% network defects, such as dangling chain ends, and 50% networked chain segments.

This paper seeks to establish whether bond breaking from the S3M process has some selectivity between mono/di/polysulfidic bonds. The cross-link densities of ground tire rubber (GTR) with increasing numbers of solid-state shear milling cycles (1–4) were established by swelling in toluene. The sulfur bond ratios of these GTRs were measured, following the process from Choi and Kim.^38^ This will allow both the S3M process itself and the revulcanization process to be modified to provide greater control of the properties of the recycled rubber. GTR that had been through four S3M cycles was also blended with virgin natural rubber (vNR), and different sulfur/accelerator ratios were applied to reintroduce specific sulfur bond types. Finally, the overall effect of cross-link densities and sulfur bond ratios of these recycled blends was evaluated by tensile testing. By establishing how devulcanization affects recyclate chemistry, new reprocessing strategies may be created to allow recycled rubber blends to more closely match the properties of virgin materials.

## 2 Experimental Section

### 2.1 Materials

Preshredded ground tire rubber was obtained from recycling facilities in China.

Zinc oxide reagent grade (ZnO), stearic acid 95% (SA), sulfur, tetramethyl thiuram disulfide 97% (TMTD), and 2-propanethiol ≥97% were procured from Sigma-Aldrich. *N*-cyclohexyl-2-benzothiazole sulfenamide (CBS) was procured from Santa Cruz Biotechnology. Hexylamine 99% was procured from Thermo Scientific. Cyclohexanethiol >98% was procured from TCI Chemical. Tetrahydrofuran (THF) was procured from Fisher Scientific. Toluene was procured from VWR Chemical. Virgin natural rubber (grade TSR 10) was obtained from Resinex (UK). Carbon black grade N330 was received from Cabot.

### 2.2 Solid-State Shear Milling

The materials were passed through the solid-state shear milling (S3M) process to obtain a homogeneous rubber powder, as described by Xu et al.^23^ The S3M process reduces the size and cross-link density of the materials with each cycle. In this case, 1–4 S3M passes were used to produce powders GTR1–4 correspondingly. This devulcanization process was performed at Sichuan University, and the materials were characterized as received at the University of Bradford.

### 2.3 Reprocessing

70 phr GTR4 was blended with 30 phr virgin natural rubber (vNR). To this compound, 13.5 phr carbon black (CB) grade N330 was added. Two different cure packages were added as described below. The cure packages were named according to their accelerator (Acc.) to sulfur (S) ratio, where the semiefficient system is referred to as “standard Acc./S” (giving material code NG4-S), and the conventional cure package is referred to as “low Acc./S” (giving material code NG4-L). The compositions of the prepared materials are listed in Table 1.

**Table 1:** Prepared Materials

Code	Material	No. of S3M cycles	Cure package
GTR1	S3M-recycled powder	1	-
GTR2	S3M-recycled powder	2	-
GTR3	S3M-recycled powder	3	-
GTR4	S3M-recycled powder	4	-
NG4-S	vNR/GTR4 30/70 blend	4	standard Acc./S
NG4-L	vNR/GTR4 30/70 blend	4	low Acc./S

Cure packages: *In each case, the curative related to the vNR was added at full dose, whereas the curative for GTR4 was added at half dose, as indicated.

“Standard A/S” – *3/1.5 phr (vNR/GTR) ZnO, 1/0.5 phr SA, 2/1 phr S, 1.5/0.75 phr CBS, 1.5/0.75 DOTG

“Low A/S” – 3/1.5 phr (vNR/GTR) ZnO, 1/0.5 phr SA, 2.5/1.25 phr S, 0.5/0.25 phr CBS, 0.5/0.25 DOTG

The blended compounds were compression molded using a Fontijne LabEcon300. The temperature and pressure were 150 °C and 10 MPa in all cases; the time was selected according to the optimum cure time for each compound as determined by rheology.

### 2.4 Characterization

#### 2.4.1 Swelling

Organic additives were removed from 15 g of each GTR via Soxhlet extraction with tetrahydrofuran (THF) and toluene for 2 and 3 days, respectively. Samples were dried for 2 days under vacuum, and the weight of the extracted GTR was measured. Total cross-link density was measured via the equilibrium swelling method. Each GTR powder was swollen in toluene until equilibrium (3–5 days); these samples were then lightly dried using a paper towel and weighed. The cross-link density (*χ*_c_) of a swollen polymer network was calculated according to the Flory–Rehner equation (eq 1).^39^


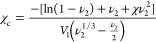
1

where χ is the polymer–solvent interaction parameter, also referred to as the solubility parameter (in this case, between GTR and toluene). *V*_1_ is the molar volume of the swelling solvent, 106.3 cm^3^/mol. *ν*_2_ is the volume fraction of the cross-linked polymer and is obtained by the following equation (eq 2) based on mass and density.


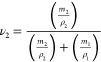
2

where *m*_1_ and *m*_2_ are the solvent and specimen weights at equilibrium swelling, respectively. ρ_1_ and ρ_2_ are the densities of the swelling solvent and unswollen GTR, respectively. In this case, ρ_1_ and ρ_2_ are 0.865 and 0.96 g/cm^3^, respectively. The solvent–rubber interaction parameter was selected as 0.393 for toluene/natural rubber.^40^

#### 2.4.2 Chemical Probes

To selectively cleave specific sulfur cross-links, chemical probe solutions were utilized, as described by Choi and Kim.^38^ Chemical probe solution 1 (CP1), for the cleavage of polysulfidic cross-links, was made up of 0.4 M propane-2-thiol and 0.4 M hexylamine in toluene. For the cleavage of di- and remaining polysulfidic cross-links, chemical probe solution 2 (CP2) was made up of 2 M cyclohexanethiol and 4 M hexylamine in toluene.

For the cleavage of polysulfidic cross-links, GTR samples were preswollen in toluene at room temperature for 24 h before being treated with CP1 for 6 h at room temperature under an inert atmosphere. For the cleavage of di- and polysulfidic cross-links, GTR samples were then treated with CP2 for 24 h at room temperature under an inert atmosphere.

Following each chemical probe treatment, samples were immediately soaked in toluene for 24 h, then soaked in fresh toluene for a further 24 h. The weights of each swollen sample were measured, and the total cross-link densities were calculated using eq 1, as described previously. An average was taken over three samples.

#### 2.4.3 Sol Fraction

The sol fraction is the soluble part of the polymer network that becomes liberated upon swelling in a solvent. As highlighted by Verbruggen et al.,^20^ the sol–gel analysis for devulcanized (selectively de-cross-linked) rubber is partly dependent upon the soluble fraction in the untreated vulcanizate, *s*_i_ as well as the cross-link index before treatment *γ*_i_. The final sol fraction (*s*_f_) for each devulcanized GTR was measured as the mass ratio , where *m*_i_ is the initial mass and *m*_f_ is the final mass before and after Soxhlet extraction with THF (48 h) and toluene (72 h). Separately, the sol fraction for each sample (using 15 g of fresh material) was also measured by direct extraction in acetone to ensure reliability.

#### 2.4.4 Rheology

Cure time (*t*_90_) was evaluated by producing torque–time graphs using an Anton-Paar Physica MCR 301 rheometer (Graz, Austria) using a 25 mm parallel plate, at 1 Hz and 1% strain, with a temperature of 150 °C. The temperature was controlled by using a p-ETD400 plate and an H-ETD400 hood. Test pieces were discs of 25.4 mm diameter and ∼1 mm thickness that were trimmed at the start of the torque–time measurement.

#### 2.4.5 Mechanical Properties

Tensile testing for rubber materials was performed according to ISO 37 with an extension rate of 500 mm/min using a 1 kN load cell. The specimens were stamped out using a modified ISO 37-2 shape cutter, which has been described previously.^28^ An average was taken over four specimens.

## 3 Results and Discussion

### 3.1 Ground Tire Rubber Recyclate

#### 3.1.1 Cross-Link Density and Type – Powder Recyclate

The cross-link densities of the GTR powders, before and after sulfur bond extraction, were measured by swelling in toluene. The change in cross-link density allows the efficacy and selectivity of the S3M process to be evaluated.

The measured cross-link density is affected by the concentration of filler, which is partly due to bound rubber^41^ and the fact that fillers do not swell. The review from Sienkiewicz et al. on GTR suggests carbon black loading in the range of 40–70 phr depending on the type of tire,^42^ and 40–50 phr is suggested by the Japan Automobile Tyre Manufacturers Association.^43^ For all of the GTR powders measured, the filler loading was considered to be 45 phr, in line with these literature values and following on from our previous work.^28^ The recycling process is not expected to have any effect on the filler loading. Verbruggen et al. demonstrated that the CB content of devulcanized rubber was unchanged when comparing the TGA of gel and sol fractions.^20^

**Figure 1:**
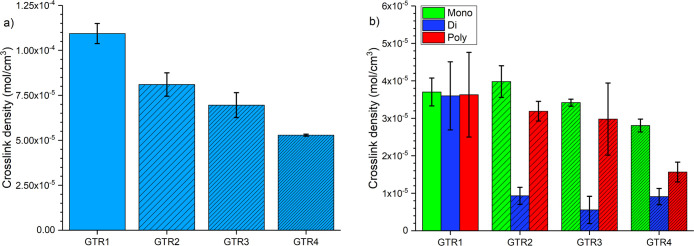
Effect of number of S3M passes on ground tire rubber: a) cross-link density, and b) sulfur composition.

With an increasing number of S3M passes, the cross-link density of the ground tire rubber reduces, as shown in Figure 1a. The powdered ground tire rubber has a cross-link density of 1.1 × 10^–4^ mol/cm^3^ after one cycle. This reduces by 26% to 7.4 × 10^–5^ mol/cm^3^ after two cycles, and after four cycles, the material has approximately half the cross-link density of GTR1.

For GTR1, the ratio of mono/di/poly-linkages is fairly even, with 3.7 × 10^–5^ mol/cm^3^ of monosulfidic cross-links, 3.6 × 10^–5^ mol/cm^3^ of disulfidic cross-links, and 3.6 × 10^–5^ mol/cm^3^ of polysulfidic cross-links, as shown in Figure 1b. Following the second devulcanization cycle, the concentration of disulfidic cross-links is dramatically reduced, whereas mono- and polysulfidic linkages appear relatively unaffected, within the margin for error. The standard deviation measured for the samples reduced with an increasing number of S3M passes. The broad range of cross-link densities and types within the waste material seemingly becomes more homogeneous with S3M passes, resulting in reduced deviation for GTR4. Overall, the S3M process gradually reduces poly- and disulfidic cross-links with little impact on monosulfidic links.

It should be considered that a proportion of the measured “mono” cross-links may be carbon–carbon links (formed by peroxide vulcanization or other means), since the formulation of the original GTR recyclate is unknown and these cannot be removed by the chemical probes utilized. However, as highlighted by Valentin et al., tire manufacture is mainly based on sulfur chemistry.^4^ They also suggest that the breakage of monosulfidic linkages may not be possible without some damage to the rubber backbone, which is undesirable. Their previous work on GTR (2100 μm particle size truck tire recyclate) found the residual cross-links to contain 7% poly-, 55% di-, and 38% mono/carbon linkages.^4^ It is worth highlighting that there is a degree of uncertainty between short polysulfidic linkages and disulfidic linkages because the chemical probes are not perfectly selective.^38^

It is noteworthy that moving from GTR3 to GTR4, the proportion of disulfidic cross-links apparently increases, while there is a substantial drop in polysulfidic links. This increase in disulfidic linkages could come from polylinkages that have been broken and reformed with shorter lengths, as is known to happen in the vulcanization of virgin rubbers. Equally, moving from GTR1 to GTR2, where the number of disulfidic links rapidly drops, the number of monosulfidic links appears to increase (although the change in monocross-links is within error). Overall, the solid-state shear milling process shortens sulfur linkages, while also reducing their overall concentration; a proportion of the bonds are broken, of which some reform shorter links. The broken cross-links that do not reform shorter links exist as radicals, which may be present in the devulcanized powder for long periods or react with atmospheric oxygen, as demonstrated by our previous work.^29^

### 3.2 Sol Fraction and Horikx Plot

Sol fraction is often used as an indicator for devulcanization, since cross-linking of the rubber chains leads to the formation of a 3D network that is insoluble in solvents. Therefore, in the cases of recycling and devulcanization, the sol fraction increases as the polymer network is broken down. For reclaimed materials (where there is little selectivity for S–S cleavage compared with C–S and C–C cleavage), the sol fraction increases more rapidly with a reduction in cross-link density than for devulcanized materials.^20^ In the case of pure sulfur bond cleavage, the sol fraction only starts to sharply increase when the cross-link density is reduced by more than 80% (see Figure 3).

**Figure 2:**
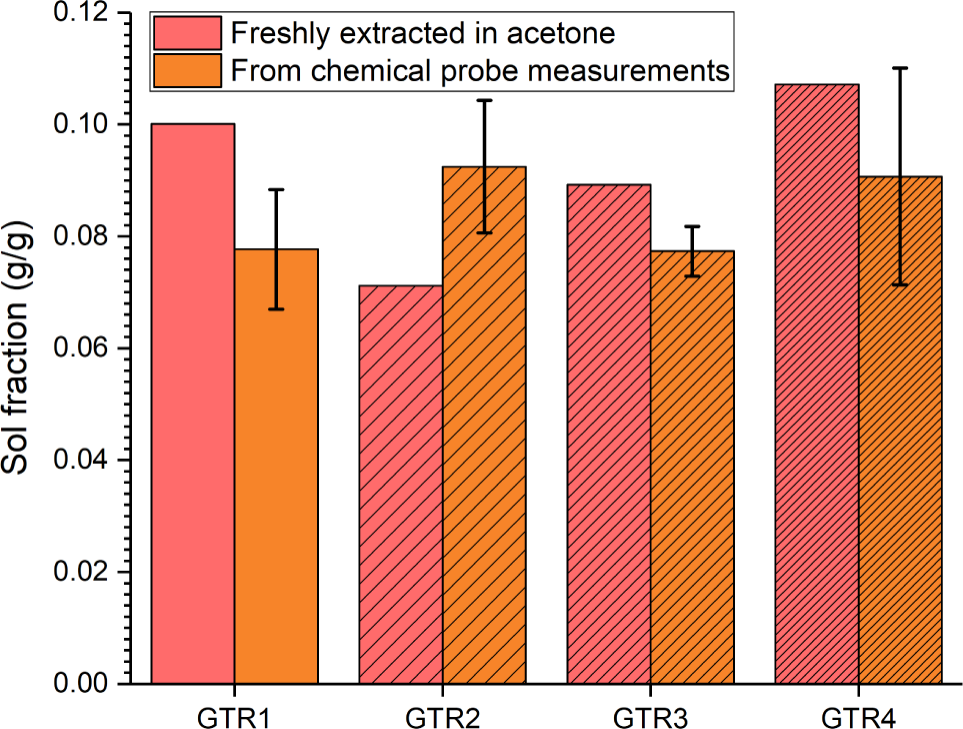
Sol fraction with an increasing number of S3M passes; left column, extracted in acetone; right column, sol content taken from chemical probe measurements.

For GTR1–4, two sets of sol fraction measurements were obtained: one using the chemical probe materials and one set from fresh samples directly extracted with acetone, as shown in Figure 2. Yet, for both sets of sol fraction measurements, there was no obvious change with increasing levels of devulcanization from GTR1 to GTR4. For both the least and most devulcanized materials (GTR1 and GTR4, respectively), the sol fraction remained constant at approximately 10%. For the sol fractions from the chemical probe measurements, the lowest measured value was recorded for GTR1, and the highest for GTR4, as would be expected. However, the significant deviation and lack of a clear trend suggest that the sol fractions were similar across specimens. This is in contrast to the cross-link density, which clearly declined with increasing S3M passes (as previously seen in Figure 1a).

**Figure 3:**
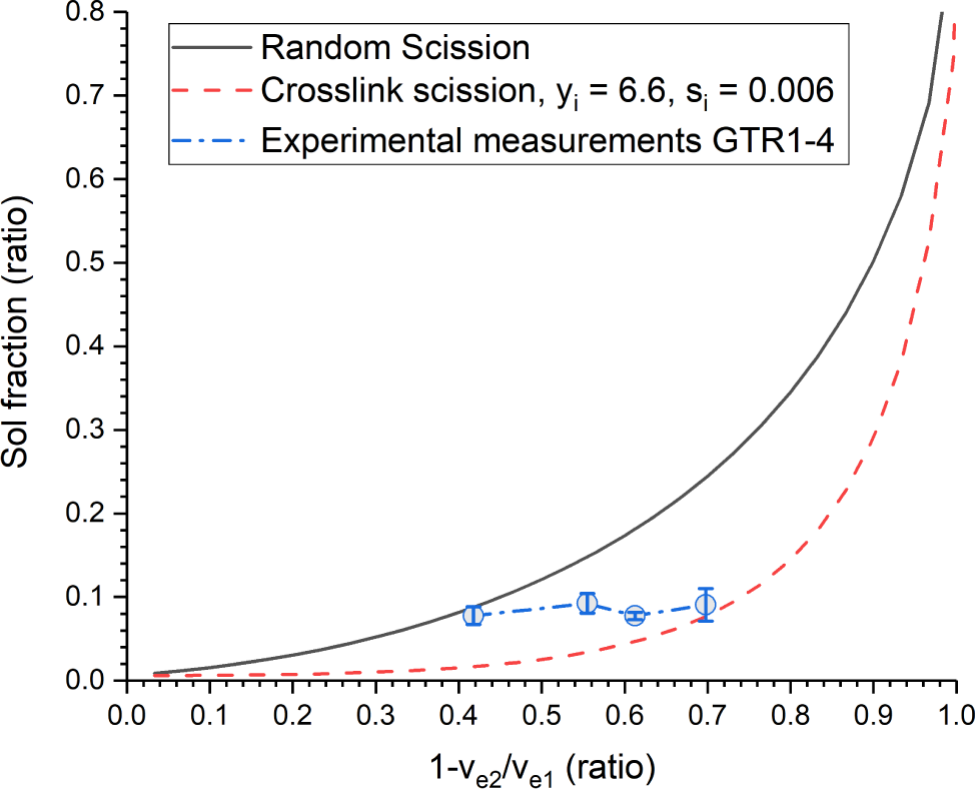
Horikx diagram showing curves for theoretical random bond scission, theoretical cross-link scission, and experimental results for GTR1–4.

Considering the Horikx diagram, which is often used to suggest selectivity between sulfur cross-link breakage (devulcanization) and random bond scission (reclamation), it is desirable to have decreasing cross-link density with relatively little change in the sol fraction. In this respect, the indication that the sol fraction is broadly similar between GTR1 and GTR4 positively suggests S3M selectivity for devulcanization. However, in this case, there are some limitations for the Horikx plot, given in Figure 3, which will be discussed.

For the reduction in the cross-link density of the GTR materials (-*v*_e2_/*v*_e1_), the cross-link density of the original waste product was assumed to be 3 × 10^–4^ mol/cm^3^, which is also the point at which the sol fraction of the rubber network is approximately 0.^45^ For the theoretical curves, cross-link density was gradually reduced and the sol fraction was calculated according to the equations described by Horikx,^17^ and verified by Verbruggen.^20^ In line with Verbruggen’s results for natural rubber, the initial cross-link index was taken as 6.6. The initial soluble fraction was taken as 0.6% in accordance with Horikx.^17,20^

The experimental results for GTR1–4 sit in between the curves for random scission and selective cross-link scission, as shown in Figure 3. However, the gradient of the experimental results is closest to the gradient in the early stages of cross-link scission. It is worth highlighting that the received GTR is already partly broken down, with an initial particle size of the order of millimeters. It is likely that the industrial grinding processes used to break down tire waste most closely matched random scission, which resulted in a significant sol fraction for the received GTR. The high sol fraction for GTR1 is therefore attributed to industrial processing of the as-received GTR before S3M takes place.

As the number of S3M passes increases, the curve mimics the results for selective cross-link scission, after having started at a higher initial sol fraction. This result, along with the minor reduction in monosulfidic cross-links seen in Section 3.1.1, indicates a selective devulcanization (rather than reclamation) process. However, there are limitations in the estimates of the initial cross-link density, initial and final cross-link index, and initial sol fraction. Since the GTR is a mixture of tire materials, it is difficult to estimate the initial cross-link density and cross-link index. Additionally, Verbruggen et al. note that physical entanglements make up a large proportion of the cross-link density as measured by swelling^20^ and it will also be affected by the residual filler fraction. This means that the measured cross-link density after devulcanization from swelling is relatively high. Other authors have measured cross-link density by DQ-NMR to mitigate these problems; however, this often requires measuring the cross-link density of the sol rather than the gel fraction.^4^ In their study, after complete cleavage of poly- and dicross-links (which accounted for 62% of total cross-links in their end-of-life tire sample), the sol fraction was close to 50% in toluene/acetone.^4^ They add that the proportion of monosulfidic cross-links is the limiting factor in achieving a truly selective devulcanization, as found here. As previously introduced, it is difficult to break monosulfidic cross-links (C–S bonds), since they have higher bond energy than S–S.

### 3.3 Blended vNR/GTR

The devulcanized GTR powder can be blended with virgin rubber to process the material into a compound that can be cured. In this case, 70 phr of GTR4 was blended with 30 phr of virgin natural rubber (vNR) on a two-roll mill. The measured cross-link density of GTR4 is substantially lower than what may be expected from a cured CB-filled rubber. It is therefore necessary to add curatives to the vNR/GTR blend in order to successfully revulcanize the material.

Two different cure packages were added to the blend in order to evaluate whether the curatives could be tailored to reintroduce specific cross-link types. This was done by varying the ratio of sulfur/accelerator only. Varying the type of curatives^3^ or cure time^37^ may also be effective but was outside the scope of this work. The prepared vNR/GTR4 blends (NG4) are named NG4-S and NG4-L, referring to a standard (semiefficient) accelerator/sulfur ratio and a low accelerator/sulfur ratio, respectively. Carbon black was also added to maintain the original concentration of CB after the addition of vNR. The effect of fillers on recycled rubber blends was discussed in our previous work.^28^

#### 3.3.1 Rheology

**Figure 4:**
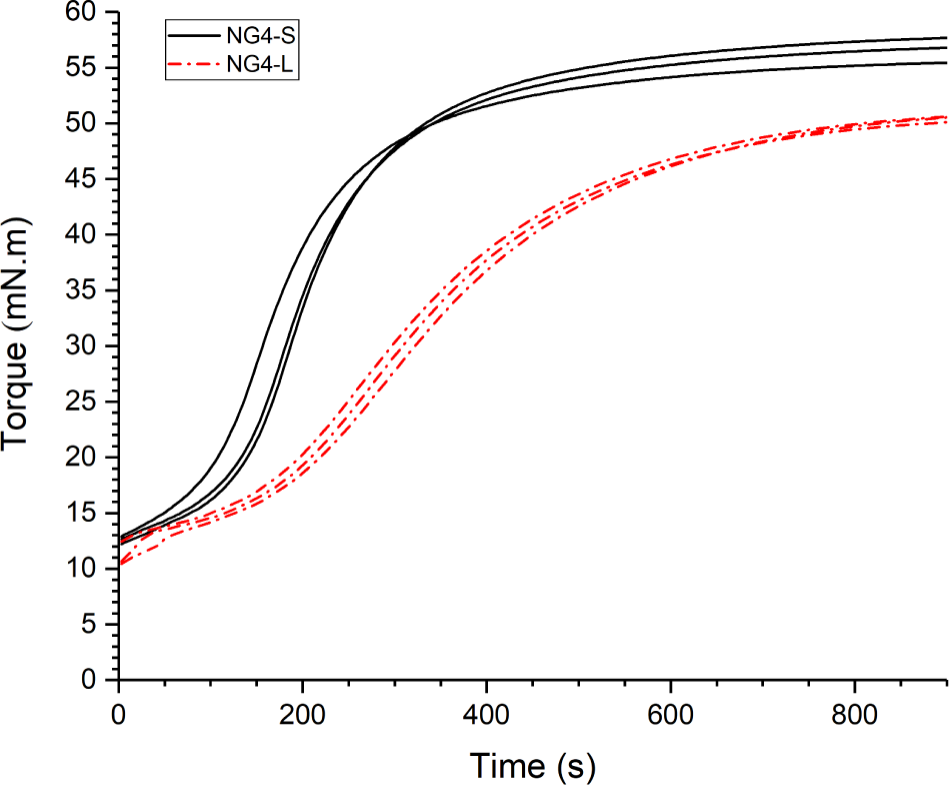
Torque–time curves (three repeats) for vNR/GTR4 blends, comparing standard and low Acc./S cure packages.

Torque–time tests were performed to measure the cure time for the prepared GTR4/vNR blends and assess their change in torque throughout vulcanization. We previously found that the optimum cure time (typically taken as the time to reach 90% of the maximum torque, *t*_90_) for high recyclate concentration blends can be much longer than for virgin natural rubber.^28^ Sulfur has been found to migrate from the matrix to ground rubber particles.^46,47^ This is accompanied by a migration of the accelerator from the recyclate to the matrix. However, this reduces cure time for virgin rubber matrices, where GTR is large in size and low in concentration. Yet, for these high recyclate content blends, where the small devulcanized rubber particles form the major constituent of the matrix, the inverse effect on the cure rate is seen. This may be a diffusion rate effect, where the residual cross-links and dangling chain ends of the devulcanized GTR result in low mobility of sulfur and accelerators, meaning the minor virgin rubber constituent cures slowly. The cure rate is also substantially affected by the sulfur/Acc. ratio for these blends. At low Acc./s, the cure rate is reduced compared with a semiefficient cure package, as shown in Figure 4. Seemingly, the cure rate is accelerator limited. Kim et al. have previously reported that bonding between vulcanized and unvulcanized rubber is most dependent on the accelerator.^48^

The difference in the shape of the torque–time curves for the two cure packages can be seen in Figure 4. The scorch time and initial torque of the cure packages were found to be very similar; however, the cure time and overall change in torque varied significantly, as shown in Table 2. The change in torque is taken as a measure of cross-link density, which suggests that the “standard” cure package produced the greatest number of cross-links. Cross-link density affects mechanical properties; therefore, the 10% increase in δtorque for S-CP may have a measurable effect on tensile strength and elongation at break. The “standard” CP (NG4-S) also showed the shortest cure time, which is considered desirable due to reduced production time and energy costs.

**Table 2:** Torque Values and Cure and Scorch Times for vNR/GTR4 Blends Using Standard and Low Acc./S Cure Packages

Material	Min torque (mN m)	Max torque (mN m)	δtorque (mN m)	Cure time (s)	Scorch time (s)
NG4-S	12.8 ± 0.3	56.7 ± 0.8	43.9 ± 1.1	408 ± 9	44 ± 4
NG4-L	11.5 ± 0.9	50.4 ± 0.2	38.9 ± 0.8	605 ± 8	40 ± 15

#### 3.3.2 Cross-Link Density and Type – vNR/GTR Blends

**Figure 5:**
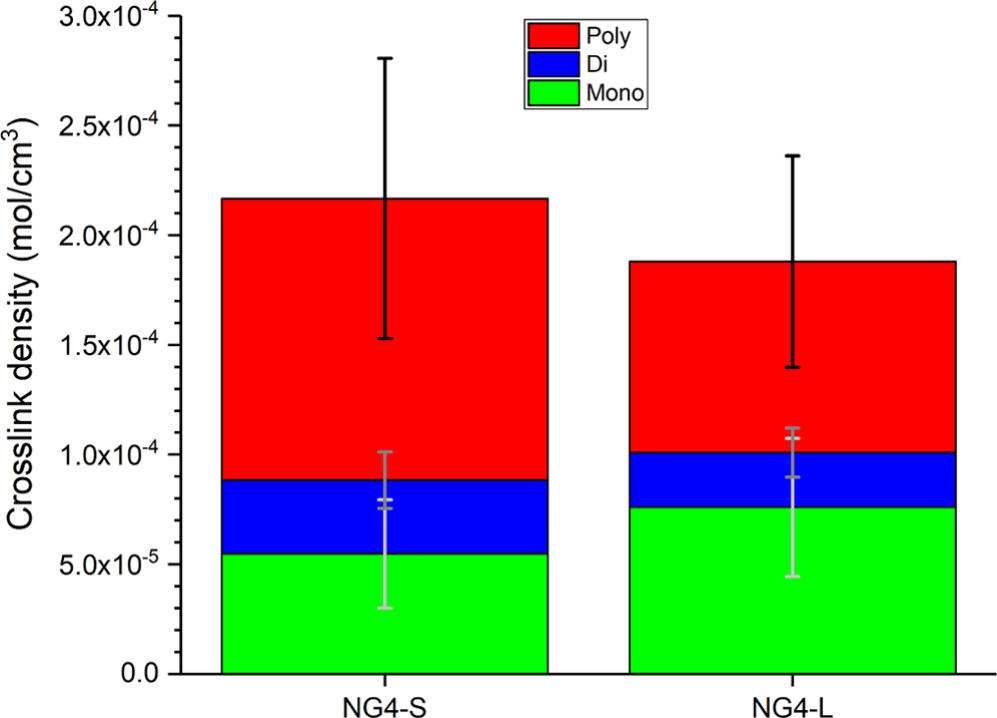
Cross-link density of vNR/GTR 30/70 blends, after compounding and revulcanization, showing two different cure packages (where vG4-L has a 0.25 accelerator/sulfur ratio, and vG4-S has a 0.75 accelerator/sulfur ratio) – measured between Soxhlet extractions as described in Section 2.4.2.

**Figure 6:**
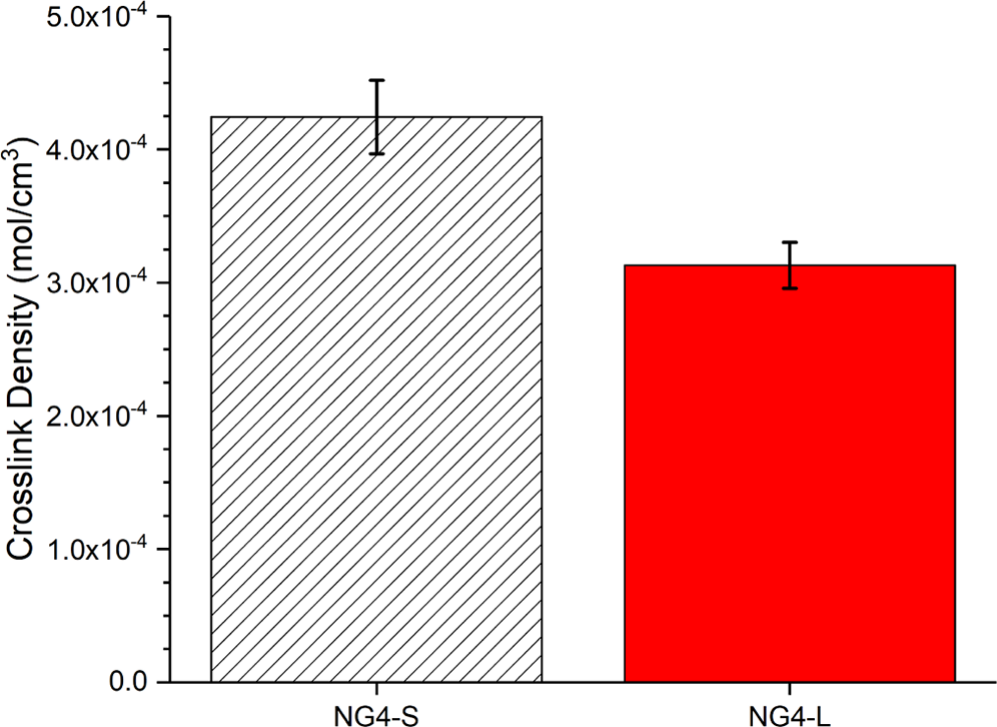
Cross-link density of NG4, as measured by basic swelling tests in toluene (no extraction), comparing standard and low Acc./S.

The cross-link density and sulfur rank of the two recycled blends were measured to evaluate how the differing cure packages affected revulcanization. For samples measured under Soxhlet extraction with chemical probes (as described in Section 2.4.2), the measured cross-link densities of the 30/70 vNR/GTR blends were 1.88 and 2.17 (× 10^–4^ mol/cm^3^) for NG4-L and NG4-S, respectively, as shown in Figure 5. Yet, the deviation across the three repeats was greater than the margin between the two compounds. Therefore, no significant difference between the two cross-link densities can be suggested. However, for cured samples that were directly swollen in toluene, a measurable difference in cross-link density was obtained, as highlighted in Figure 6. The samples without extraction show higher overall cross-link density than those with extraction. It is suggested that the Soxhlet extraction process with multiple chemical probe steps reduces physical entanglements, thereby reducing the measured cross-link density. Previously, it has been suggested that physical entanglements make up a significant proportion of the total measured cross-link density.^4,49,50^ In this case, NG4-S shows a significantly greater cross-link density than NG4-L, which is in line with the difference in δtorque from rheological measurements. This indicates that physical entanglements/network defects in revulcanized materials may be significantly affected by the chosen cure package.

Although the overall cross-link densities of the two differing cure packages were designed to be similar, the ratio of mono/di/polysulfidic cross-links was intended to be different. However, the differences in concentrations of poly-, di-, and monosulfidic cross-links were within error. As such, control of revulcanization proves to be more difficult than that of the virgin equivalent, which can be attributed to the radicals and chemical intermediaries present in recycled materials.

The cross-link density measured with Soxhlet extraction to determine sulfur rank (Figure 5) shows a much lower total apparent cross-link density than for the samples that have been directly measured in toluene without any extraction steps (Figure 6), which may be attributed to differences in physical entanglements and filler effects.

After devulcanization, mono- and disulfidic linkages were measurably different across differing numbers of S3M cycles, as demonstrated in Figure 1b. GTR4, which was selected for recompounding, was measured to have sulfur cross-link concentrations of 6.8, 2.0, and 3.3 (× 10^–4^ mol/cm^3^) for mono-, di- and poly-, respectively. For NG4-L, these values increased by 12, 25, and 162% for mono-, di-, and polysulfidic linkages, respectively. The overall cross-link density increased by 55% following revulcanization, which was primarily from the increase of polysulfidic cross-links. Similarly, for NG4-S, which represents a semiefficient sulfur cure package, the increase in cross-link density was predominantly from polysulfidic linkages (286% increase). Monosulfidic linkages were measured to have decreased by 19%; however, the measurement was within error, and it is expected that the true number of cross-links was similar for the devulcanized material and the compounded and revulcanized material. Increases in disulfidic cross-links were also within deviation for both compounded materials compared with the devulcanized powder.

The strong preference of the revulcanized material to form polysulfidic cross-links differs from virgin compounds. Particularly, in the case of the compound with a semiefficient cure package, where polysulfidic linkages initially formed during vulcanization would be expected to reduce in length (toward di- and mono-bonds) as vulcanization progressed toward the optimum cure time.^2,51^ This highlights differences between virgin rubber and recycled blends that must be considered when trying to recycle end-of-life rubber products. If the same cross-link density and sulfur rank as the original product cannot be matched, then properties cannot be expected to be similar. This favorability for the formation of polysulfidic cross-links may be beneficial, first because devulcanization processes also have some favorability for polysulfidic cleavage, and second because polysulfidic cross-links could be manipulated by postcure treatments or extended cure time.

Formela et al. previously investigated the effect of different curatives and Acc./S ratios on revulcanization for reclaimed rubber (without blending in the virgin material).^52^ Increases in rheological torque upon curing and cross-link density were highest for TMTD-accelerated vulcanization. It is noteworthy that TMTD promotes the formation of di- and monosulfidic cross-links as vulcanization progresses. Herein, the combination of CBS (which was concluded to be the best accelerator in terms of processing and mechanical properties by Formela et al.) and guanidine accelerator (which gave the best elongation at break) clearly favored polysulfidic linkages, regardless of the Acc./S ratio. Comparing the sulfur rank of recycled blends with that of GTR1, balancing the sulfur rank and increasing cross-link density by further addition of mono- and disulfidic cross-links, may be desirable. Mangili et al. previously compounded virgin natural rubber with a variety of devulcanized GTRs, substituting 5 phr of vNR for 10 phr of recyclate without changing the cure package. In all cases, the cross-link density of the recycled blend was reduced compared with the virgin material (although by less than 10%), having a negative effect on mechanical properties.^53^

#### 3.3.3 Tensile Properties

Higher cross-link density should result in higher stiffness; therefore, based on the rheological and swelling results, NG4-S may be expected to produce a higher modulus at 100% strain (M100) compared with NG4-L. However, this will also depend on the length of cross-links (cross-link type ratio). Previous results have demonstrated that the elongation at break (EB) also depends on the cross-link structure.^54^

**Figure 7:**
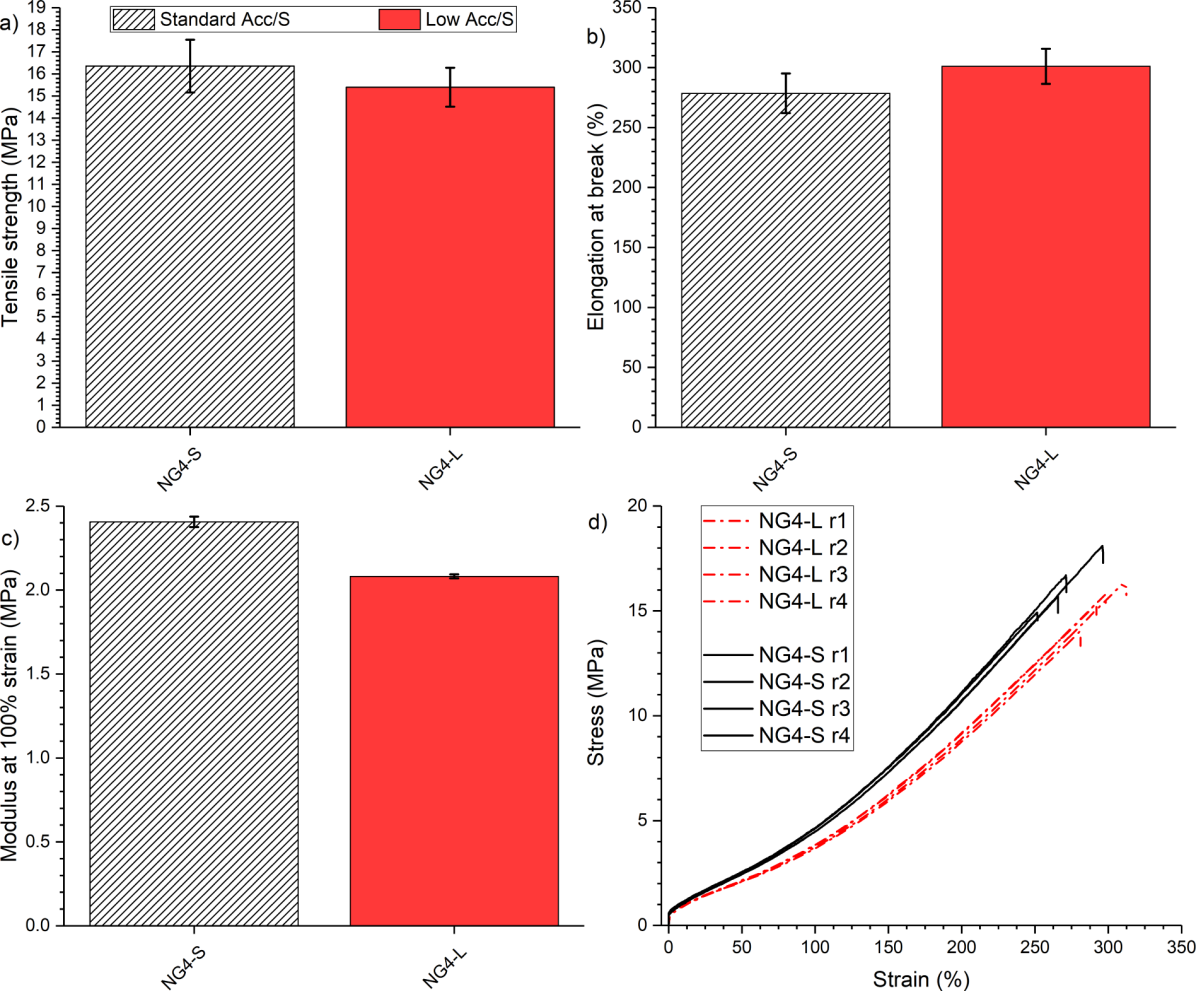
Tensile properties for the NG4 blends with standard and low Acc./S cure packages: a) tensile strength, b) elongation at break, c) modulus at 100% strain, and d) stress–strain curves showing four repeats for each material.

The tensile strengths of both prepared (30/70) vNR/GTR4 blends, NG4-S and NG4-L, are similar at approximately 16 MPa, as shown in Figure 7a. This tensile strength is similar to those reported previously for equivalent vNR/GTR1 blends and significantly below the vNR equivalent of ∼26 MPa reported in our previous work.^28^ The elongation at break for both recycled blends is approximately 300%, with the low Acc./S and standard Acc./S achieving 301 and 279%, respectively, as shown in Figure 7b. Comparing the cure packages, the low accelerator/S ratio resulted in higher elongation at break but reduced modulus, which can be attributed to the difference in cross-link densities. Previously, Rattanasom et al. found hardness, modulus, and tensile strength all to increase for a conventional cure system (low Acc./S) compared with an efficient cure system (high acc./S) for recycled rubber blends.^55^ This was attributed to higher cross-link density, which resulted in a decreased elongation at break.

Comparing different curatives, Formela et al. found CBS and TBBS to give the highest tensile strength for revulcanized rubber, while TMTD gave the greatest modulus and DPG gave the highest elongation at break.^52^ However, across all of their prepared materials, the EB and UTS remained below 250% and 8 MPa, which limits possible applications. The authors also found increasing the accelerator-to-sulfur ratio (from 1:2 to 2:1) increased elongation at break and reduced the modulus. The same result is replicated here for accelerator/S ratios of 3:2 (standard) and 2:5 (low). Yet, the results could be attributed to the reduced cross-link density for the low Acc./S ratio (in this case, NG4-L), as demonstrated in Figure 6. Therefore, it remains to be seen how sulfur rank can be controlled during revulcanization to influence properties similar to those of virgin materials. This is important because product properties are controlled through different accelerators and cure packages, so a company finding that recycled rubber has unexpected properties may attribute it to the feedstock when part of the reason may be the difference between vulcanization (of virgin material) and revulcanization (of recyclate). This highlights an important area for further research to generate the acceptance of devulcanized rubber.

## 4 Conclusions

It was found that mechanical devulcanization by solid-state shear milling selectively broke poly- and disulfidic cross-links with little effect on monosulfidic concentration. This selectivity for specific sulfur bonds rather than random scission was backed up by sol/gel measurements, which showed little change in the sol fraction. The two cure packages used to revulcanize recycled rubber/virgin rubber blends both showed high selectivity for the formation of polysulfidic cross-links, despite significant differences in the sulfur/accelerator ratio. The “standard” semiefficient cure package with an S/Acc. ratio of 0.75 saw the greatest increase in overall cross-link density. However, this may have been due to physical entanglements more so than chemical cross-link differences, as highlighted by the differences in regular swelling versus Soxhlet extraction. Unsurprisingly, the material with the higher cross-link density showed higher tensile strength and modulus but lower elongation at break. The results suggest that the sulfur/accelerator ratio alone is not sufficient to alter sulfur rank for recycled rubber blends, unlike with virgin rubber. For true circular recycling of products like tires, seals, etc., the sulfur rank and cross-link density of the original product and the postrecycled material should match. Our results emphasize the need for further investigation into revulcanization mechanisms so that recycled rubber chemistry can be controlled in a similar manner to virgin rubber, unlocking the market for sustainable rubber products.

## Data Availability

The data required to reproduce these findings is available to download from Mendeley data: DOI: 10.17632/d87tmr6td3.2.

## References

[ref1] Gent A. N. Rubber Elasticity: basic Concepts and Behavior; 4th ed., Elsevier Inc., 2013. DOI: 10.1016/B978-0-12-394584-6.00001-7.

[ref2] Akiba M.; Hashim A. S. Vulcanization and crosslinking in elastomers. Prog. Polym. Sci. 1997, 22, 475–521. 10.1016/S0079-6700(96)00015-9.

[ref3] Ghosh P.; Katare S.; Patkar P.; Caruthers J. M.; Venkatasubramanian V.; Walker K. A. Sulfur Vulcanization of Natural Rubber for Benzothiazole Accelerated Formulations. Rubber Chem. Technol. 2003, 76, 592–693. 10.5254/1.3547762.

[ref4] Valentín J. L.; Pérez-Aparicio R.; Fernandez-Torres A.; Posadas P.; Herrero R.; Salamanca F. M.; Navarro R.; Saiz-Rodríguez L. Advanced characterization of recycled rubber from end-of-life tires. Rubber Chem. Technol. 2020, 93, 683–703. 10.5254/rct.20.79963.

[ref5] Liu Z.; Li X.; Xu X.; Wang X.; Dong C.; Liu F.; Wei W. Devulcanizaiton of waste tread rubber in supercritical carbon dioxide: Operating parameters and product characterization. Polym. Degrad. Stab. 2015, 119, 198–207. 10.1016/j.polymdegradstab.2015.05.017.

[ref6] Molanorouzi M.; Mohaved S. O. Reclaiming waste tire rubber by an irradiation technique. Polym. Degrad. Stab. 2016, 128, 115–125. 10.1016/j.polymdegradstab.2016.03.009.

[ref7] Joseph A. M.; Madhusoodhanan K. N.; Alex R.; George B. Stable free radical-assisted mechanical devulcanization of carbon black-filled natural rubber vulcanizates. Rubber Chem. Technol. 2018, 91, 469–491. 10.5254/rct.18.81633.

[ref8] De Sousa F. D. B.; Zanchet A.; Scuracchio C. H. From Devulcanization to Revulcanization: Challenges in Getting Recycled Tire Rubber for Technical Applications. ACS Sustainable Chem. Eng. 2019, 7, 8755–8765. 10.1021/acssuschemeng.9b00655.

[ref9] Diaz R.; Colomines G.; Peuvrel-Disdier E.; Deterre R. Thermo-mechanical recycling of rubber: Relationship between material properties and specific mechanical energy. J. Mater. Process. Technol. 2018, 252, 454–468. 10.1016/j.jmatprotec.2017.10.014.

[ref10] Zhang X.; Lu C.; Liang M. Properties of natural rubber vulcanizates containing mechanochemically devulcanized ground tire rubber. J. Polym. Res. 2009, 16, 411–419. 10.1007/s10965-008-9243-x.

[ref11] Ghorai S.; Bhunia S.; Roy M.; De D. Mechanochemical devulcanization of natural rubber vulcanizate by dual function disulfide chemicals. Polym. Degrad. Stab. 2016, 129, 34–46. 10.1016/j.polymdegradstab.2016.03.024.

[ref12] Tapale M.; Isayev A. I. Continuous ultrasonic devulcanization of unfilled NR vulcanizates. J. Appl. Polym. Sci. 1998, 70, 2007–2019. 10.1002/(SICI)1097-4628(19981205)70:10<2007::AID-APP17>3.0.CO;2-D.

[ref13] Dukhuis K. A. J.; Babu I.; Lopulissa J. S.; Noordermeer J. W. M.; Dierkes W. K. A mechanistic approach to EPDM devulcanization. Rubber Chem. Technol. 2008, 81, 190–208. 10.5254/1.3548204.

[ref14] Saiwari S.; Dierkes W. K.; Noordermeer J. W. M. Devulcanization of whole passenger car tire material. KGK, Kautsch. Gummi Kunstst. 2013, 66, 20–25.

[ref15] Sabzekar M.; Chenar M. P.; Mortazavi S. M.; Kariminejad M.; Asadi S.; Zohuri G. Influence of process variables on chemical devulcanization of sulfur-cured natural rubber. Polym. Degrad. Stab. 2015, 118, 88–95. 10.1016/j.polymdegradstab.2015.04.013.

[ref16] Mangili I.; Collina E.; Anzano M.; Pitea D.; Lasagni M. Characterization and supercritical CO2 devulcanization of cryo-ground tire rubber: Influence of devulcanization process on reclaimed material. Polym. Degrad. Stab. 2014, 102, 15–24. 10.1016/j.polymdegradstab.2014.02.017.

[ref17] Horikx M. M. Chain scissions in a polymer network. J. Polym. Sci. 1956, 19, 445–454. 10.1002/pol.1956.120199305.

[ref18] Seghar S.; Asaro L.; Rolland-Monnet M.; Aït Hocine N. Thermo-mechanical devulcanization and recycling of rubber industry waste. Resour. Conserv. Recycl. 2019, 144, 180–186. 10.1016/j.resconrec.2019.01.047.

[ref19] Saiwari S.; van Hoek J. W.; Dierkes W. K.; Reuvekamp L. E. A. M.; Heideman G.; Blume A.; Noordermeer J. W. M. Upscaling of a batch de-vulcanization process for ground car tire rubber to a continuous process in a twin screw extruder. Materials 2016, 9, 724 10.3390/ma9090724. 28773843 PMC5457053

[ref20] Verbruggen M. A. L.; Van Der Does L.; Dierkes W. K.; Noordermeer J. W. M. Experimental validation of the Charlesby and Horikx models applied to de-vulcanization of sulfur and peroxide vulcanizates of NR and EPDM. Rubber Chem. Technol. 2016, 89, 671–688. 10.5254/rct.16.83776.

[ref21] Myhre M.; Saiwari S.; Dierkes W.; Noordermeer J. Rubber Recycling: Chemistry, processing, and applications. Rubber Chem. Technol. 2012, 85, 408–449. 10.5254/rct.12.87973.

[ref22] Sripornsawat B.; Saiwari S.; Pichaiyut S.; Nakason C. Influence of ground tire rubber devulcanization conditions on properties of its thermoplastic vulcanizate blends with copolyester. Eur. Polym. J. 2016, 85, 279–297. 10.1016/j.eurpolymj.2016.10.031.

[ref23] Xu X.; Wang Q.; Kong X.; Zhang X.; Huang J. Pan mill type equipment designed for polymer stress reactions: Theoretical analysis of structure and milling process of equipment. Plast., Rubber Compos. Process. Appl. 1996, 25, 152–158.

[ref24] Zhang X. X.; Lu C. H.; Liang M. Devulcanisation of natural rubber vulcanisate through solid state mechanochemical milling at ambient temperature. Plast. Rubber Compos. 2007, 36, 370–376. 10.1179/174328907X237584.

[ref25] Sun F.; Bai S.; Wang Q. Structures and properties of waste silicone cross-linked polyethylene de-cross-linked selectively by solid-state shear mechanochemical technology. J. Vinyl Addit. Technol. 2019, 25, 149–158. 10.1002/vnl.21636.

[ref26] Wu H.; Sun X.; Zhang W.; Zhang X.; Lu C. Effect of solid-state shear milling on the physicochemical properties of thermally conductive low-temperature expandable graphite/low-density polyethylene composites. Compos. Composites, Part A 2013, 55, 27–34. 10.1016/j.compositesa.2013.08.009.

[ref27] Wang Q.; Bai S.; Yang S.; Sun F.; Recycling Waste Polymer Materials by Solid-State Shear Milling Technology. 2016. RCS Publishing.

[ref28] Innes J. R.; Siddique N.; Hebda M.; Thompson G.; Wang X.; Coates P.; Whiteside B.; Benkreira H.; Caton-Rose P.; Lu C.; Wang Q.; Kelly A. Micromechanical modeling of devulcanized ground tyre rubber, graphene platelets, and carbon black in recycled natural rubber blends. J. Appl. Polym. Sci. 2023, 140, 1–15. 10.1002/app.54435.

[ref29] Innes J. R.; Shriky B.; Allan S.; Wang X.; Hebda M.; Coates P.; Whiteside B.; Benkreira H.; Caton-Rose P.; Lu C. H.; et al. Effect of solid-state shear milled natural rubber particle size on the processing and dynamic vulcanization of recycled waste into thermoplastic vulcanizates. Sustainable Mater. Technol. 2022, 32, e00424 10.1016/j.susmat.2022.e00424.

[ref30] Markl E.; Lackner M. Devulcanization technologies for recycling of tire-derived rubber: A review. Materials 2020, 13, 1246 10.3390/ma13051246. 32164175 PMC7085078

[ref31] Rybiński P.; Janowska G. Effect of the spatial network structure and cross-link density of diene rubbers on their thermal stability and fire hazard. J. Therm. Anal. Calorim. 2014, 117, 377–386. 10.1007/s10973-014-3673-y.

[ref32] Saville B.; Watson A. A. Structural Characterization of Sulphur Vulcanized Rubber Networks. Rubber Chem. Technol. 1967, 40, 100–148. 10.5254/1.3539039.

[ref33] Hagen R.; Salmén L.; Stenberg B. Effects of the type of crosslink on viscoelastic properties of natural rubber. J. Polym. Sci., Part B: polym. Phys. 1996, 34, 1997–2006. 10.1002/(SICI)1099-0488(19960915)34:12<1997::AID-POLB5>3.0.CO;2-N.

[ref34] Ramarad S.; Khalid M.; Ratnam C. T.; Chuah A. L.; Rashmi W. Waste tire rubber in polymer blends: A review on the evolution, properties and future, Prog. Mater. Sci. 2015, 72, 100–140. 10.1016/j.pmatsci.2015.02.004.

[ref35] Zhang X. X.; Lu C. H.; Liang M. Preparation of rubber composites from ground tire rubber reinforced with waste-tire fiber through mechanical milling. J. Appl. Polym. Sci. 2007, 103, 4087–4094. 10.1002/app.25510.

[ref36] Campbell D. S.; Saville B. Current principles and practices in elucidating structure in sulfur vulcanized elastomers. In Proceedings of International Rubber Conference, 1967, IEEE, pp. 1 14

[ref37] Blow C. M.; Loo C. T. Influence of cure system concentration on crosslink structure in SBR sulphur vulcanizates. Polymer 1975, 16, 205–208. 10.1016/0032-3861(75)90055-5.

[ref38] Choi S. S.; Kim E. A novel system for measurement of types and densities of sulfur crosslinks of a filled rubber vulcanizate. Polym. Test. 2015, 42, 62–68. 10.1016/j.polymertesting.2014.12.007.

[ref39] Flory P. J.; Rehner J. Statistical mechanics of cross-linked polymer networks II. Swelling. J. Chem. Phys. 1943, 11, 521–526. 10.1063/1.1723792.

[ref40] Sheehan C. J.; Bisio A. L. Polymer Solvent Interaction Parameters. Rubber Chem. Technol. 1966, 39, 149–192. 10.5254/1.3544827.

[ref41] Brennan J. J.; Lambert D. H. Rubber-Black Interaction Influence on Cure Level of Vulcanizates. Rubber Chem. Technol. 1972, 45, 94–105. 10.5254/1.3544716.

[ref42] Sienkiewicz M.; Kucinska-Lipka J.; Janik H.; Balas A. Progress in used tyres management in the European Union: A review. Waste Manage. 2012, 32, 1742–1751. 10.1016/j.wasman.2012.05.010. 22687707

[ref43] Valentini F.; Pegoretti A. End-of-life options of tyres. A review. Adv. Ind. Eng. Polym. Res. 2022, 5, 203–213. 10.1016/j.aiepr.2022.08.006.

[ref45] Dijkhuis K. Recycling of Vulcanized EPDM-Rubber; University of Twente, 2008.

[ref46] Gibala D.; Thomas D.; Hamed G. R. Cure and mechanical behavior of rubber compounds containing ground vulcanizates: Part III. Tensile and tear strength. Rubber Chem. Technol. 1999, 72, 357–360. 10.5254/1.3538807.

[ref47] Jacob C.; De P P.; Bhowmick A. K.; De K S. Recycling of EPDM waste. I. Effect of ground EPDM vulcanizate on properties of EPDM rubber. J. Appl. Polym. Sci. 2001, 82, 3293–3303. 10.1002/app.2188.

[ref48] Kim S. W.; Park H. Y.; Seo K. H. Effects of cure systems of ground rubber and rubber matrix on their adhesion and crosslink structures. Rubber Chem. Technol. 2006, 79, 806–819. 10.5254/1.3547968.

[ref49] Schlögl S.; Trutschel M. L.; Chassé W.; Riess G.; Saalwächter K. Entanglement effects in elastomers: Macroscopic vs microscopic properties. Macromolecules 2014, 47, 2759–2773. 10.1021/ma4026064.

[ref50] Howse S.; Porter C.; Mengistu T.; Pazur R. J. Experimental determination of the quantity and distribution of chemical crosslinks in unaged and aged natural rubber, part 1: Peroxide vulcanization. Polym. Test. 2018, 70, 263–274. 10.1016/j.polymertesting.2018.07.002.

[ref51] Geyser M.; McGill W. J. Thiuram-accelerated sulfur vulcanization. III. The formation of crosslinks. J. Appl. Polym. Sci. 1996, 60, 439–447. 10.1002/(SICI)1097-4628(19960418)60:3<439::AID-APP18>3.0.CO;2-Y.

[ref52] Formela K.; Wąsowicz D.; Formela M.; Hejna A.; Haponiuk J. Curing characteristics, mechanical and thermal properties of reclaimed ground tire rubber cured with various vulcanizing systems. Iran. Polym. J. 2015, 24, 289–297. 10.1007/s13726-015-0320-9.

[ref53] Mangili I.; Lasagni M.; Anzano M.; Collina E.; Tatangelo V.; Franzetti A.; Caracino P.; Isayev A. I. Mechanical and rheological properties of natural rubber compounds containing devulcanized ground tire rubber from several methods. Polym. Degrad. Stab. 2015, 121, 369–377. 10.1016/j.polymdegradstab.2015.10.004.

[ref54] Grasland F.; Chazeau L.; Chenal J. M.; Caillard J.; Schach R. About the elongation at break of unfilled natural rubber elastomers. Polymer 2019, 169, 195–206. 10.1016/j.polymer.2019.02.032.

[ref55] Rattanasom N.; Poonsuk A.; Makmoon T. Effect of curing system on the mechanical properties and heat aging resistance of natural rubber/tire tread reclaimed rubber blends. Polym. Test. 2005, 24, 728–732. 10.1016/j.polymertesting.2005.04.008.

